# Pan-cancer analysis reveals RIPK2 predicts prognosis and promotes immune therapy resistance via triggering cytotoxic T lymphocytes dysfunction

**DOI:** 10.1186/s10020-022-00475-8

**Published:** 2022-05-04

**Authors:** Junquan Song, Runyu Yang, Rongyuan Wei, Yue Du, Pengcheng He, Xiaowen Liu

**Affiliations:** 1 Department of Gastric Surgery, Fudan University Shanghai Cancer Center, 200030 Shanghai, China; 2 Department of Hematology, The First Affiliated Hospital of Xi’an Jiaotong University, 710000 Xi’an, China; 3 Department of Oncology, Shanghai Medical College of Fudan University, 200030 Shanghai, China

**Keywords:** RIPK2, Receptor-interacting protein kinase 2, Tumor immunity, Immunotherapy, Inflammation, Biomarker

## Abstract

**Background:**

Receptor-interacting protein kinase 2 (RIPK2, also known as RIP2) was reported to be associated with bacterial infections as well as inflammatory responses. However, the role of RIPK2 in prognosis and immunotherapy response is yet to be elucidated in human pan-cancer.

**Methods:**

In this study, we investigated the expression, gene alteration landscape and prognostic value of RIPK2 in 33 cancers through various databases including Ualcan, cBioportal and Gene Expression Profiling Interactive Analysis 2 (GEPIA2). Then, the correlation between RIPK2 and immune infiltration, immune score, stromal score, and ESTIMATE score was investigated in the Cancer Genome Atlas (TCGA) and tumor immune estimation resource (TIMER) databases. Independent cohorts were utilized to explore the role of RIPK2 in tumor immunotherapy response. Furthermore, Gene set enrichment analysis (GSEA) was conducted to explore the mechanisms by which RIPK2 regulates immune therapy resistance. Single-cell RNA-seq datasets were used to analyze the expression level of RIPK2 on different immune cells. Moreover, CellMiner database was used to explore the relationship between RIPK2 expression with drug response.

**Result:**

Compared with normal tissue, tumor tissue had a higher expression level of RIPK2 in various cancers. Survival analysis showed that high expression of RIPK2 associated with poor prognosis in numerous cancers. RIPK2 was found to promote a series of immune cell infiltration and B cells, macrophages, and neutrophils were significantly positively correlated with the expression of RIPK2. Moreover, RIPK2 affected immune score, stromal score and ESTIMATE score for a wide range of cancers. In the vast majority of 33 cancers, gene co-expression analysis showed that RIPK2 was positively correlated with the expression of immune checkpoint markers, such as PDCD1 (PD-1), CD274 (PD-L1), CTLA4 and TIGIT. RIPK2 aggravated cytotoxic T lymphocyte (CTL) dysfunction and related to the poor efficacy of immune checkpoint blockade in skin cutaneous melanoma (SKCM) and kidney renal clear cell carcinoma (KIRC). High expression of RIPK2 promoted innate immunotherapy resistance and adaptive immunotherapy resistance through IL-6/JAK/STAT3 signaling, interferon-gamma response, and interferon-alpha response pathway.

**Conclusions:**

These results confirmed that RIPK2 could serve as a prognostic biomarker and promoted immune therapy resistance via triggering cytotoxic T lymphocytes dysfunction.

## Introduction

Cancer is one of the leading causes of death and the most important obstacle to long life expectancy around the world. In 2018, there was about 18.1 million new cancer cases and 9.6 million cancer deaths, putting a heavy burden on the world (Bray et al. 2018). Immunotherapies have revolutionized the field of oncology through improving the survival time of cancer patients (Waldman et al. 2020). However, cancer treatment is still unsatisfying, and it is urgent to explore pathogenesis and potential therapeutic targets.

Receptor-interacting protein kinase 2 (RIPK2, also known as RIP2) was first reported in 1998, associated with NF-κB activation and cell death (McCarthy et al. 1998). As a member of the RIPKs family, RIPK2 plays a major role in bacterial infections and inflammatory responses (He and Wang 2018). Activity of RIPK2 is essential for the bacteria-sensing receptors NOD1/2 inflammatory signaling pathway, inducing proinflammatory and antimicrobial responses, and RIPK2 inhibitors significantly inhibit the immune response of NOD1/2 (Hrdinka et al. 2018). Recent studies have shown that RIPK2 was involved in the occurrence, development, and prognosis of cancer (Li et al. 2020; Zhou et al. 2021). The silence of RIPK2 suppressed the growth of gastric cancer cells by inhibiting cell migration and inducing apoptosis through the NF-κB signaling pathway (Yang et al. 2021). Knocking down RIPK2 reduces the activity of breast cancer cells, and increases sensitivity to docetaxel, reducing lung metastasis and tumor mass in a xenograft mouse model (Singel et al. 2014). Besides, the strong expression of RIPK2 was significantly correlated to the high-risk clinical stage, and metastasis features, which predicts poor prognosis in kidney renal clear cell carcinoma (Li et al. 2021). Although the role of RIPK2 in cancer is increasingly reported, there is no systematic study of RIPK2’s function and clinical importance in human pan-cancer.

In this study, comprehensive analysis was conducted based on various databases, we visualized the expression and prognostic value of RIPK2 in pan-cancer, and investigated its relationship to immunotherapy response, as well as its possible molecular biological functions. The results showed that RIPK2 was overexpressed in multiple cancers and was negatively related to the prognosis of patients. Moreover, RIPK2 was involved in immunotherapy resistance through promoting cytotoxic T lymphocytes dysfunction. Overall, our research demonstrated the crucial role of RIPK2 in anti-tumor immune and immunotherapy response, providing new targets for cancer treatment.

## Methods

### The expression of RIPK2 in human cancer

Tumor tissue and normal tissue RNA seq data in the TPM format of 33 human cancers in the Cancer Genome Atlas (TCGA) and Genotype-Tissue Expression (GTEx) databases, which are handled uniformly by the Toil process, was downloaded from the UCSC XENA (https://xenabrowser.net/datapages/) website. First of all, we used TCGA data to compare the differences in the expression of RIPK2 in various types of cancer in the tumor immune estimation resource database (TIMER, http://timer.cistrome.org/). In addition, the sample was analyzed for gene difference expression using the Wilcoxon rank-sum test in R software. Then, we visualized the differences in gene expression with the ggplot2 package (version 3.3.3). GEPIA 2 (http://gepia2.cancer-pku.cn/) is a web-based tool which can deliver fast and customizable functionalities based on TCGA and GTEx databases (Tang et al. 2019). The “stage plot” module was selected, then we entered “RIPK2” and selected the type of cancer we focused on in “Datasets Selection” and we got the correlation between RIPK2 expression level and tumor pathological stages. TNMplotter (https://tnmplot.com/analysis/) was utilized to explore the difference of RIPK2 expression in tumor, normal, and metastatic tissues across human pan-cancer (Bartha and Gyorffy 2021). The Human Protein Atlas (HPA) database (www.proteinatlas.org) is designed to map all the human proteins in cells, tissues and organs. Tissue Atlas was used to show expression of RIPK2 protein via immunohistochemistry staining.

### Survival prognosis analysis

GEPIA2 (http://gepia2.cancer-pku.cn/) is an online platform for analyzing the TCGA and the GTEx databases, which provides customizable functions. The relationship of RIPK2 expression and clinical outcome in human cancers was investigated in “Svival analysis” module, and selected median as the group cutoff. Meanwhile, overall survival (OS) and disease-free survival (DFS) Kaplan–Meier plot and survival map were obtained from GEPIA2. Log-rank test was conducted for survival analysis. Hazard Ratio (HR) was defined as the hazard in the high RIPK2 expression group divided by the hazard in the low expression group, which was utilized to assess the influence of RIPK2 expression on patients’ survival.

### Genetic alteration analysis

The online tool cBioPortal (https://www.cbioportal.org/) integrating genetic alteration data, was used to analyze RIPK2 alteration in human cancers (Cerami et al. 2012). Through the “Cancer Type Summary” module, the RIPK2 alteration landscape of pan-cancer was depicted based on TCGA Pan-cancer Alta Studies. The RIPK2 mutation site plot was derived from “Mutation” module. To explore the relationship between RIPK2 alteration with clinical outcome of patients, we selected the Ovarian Serous Cystadenocarcinoma (TCGA, PanCancer Atlas), Sarcoma (TCGA, PanCancer Atlas), Pancreatic Adenocarcinoma (TCGA, PanCancer Atlas) and divided cases into altered group and unaltered group. The “Comparison/Survival” module was utilized to draw survival plot.

### RIPK2 and tumor immune microenvironment

TIMER database (https://cistrome.shinyapps.io/timer/) is a comprehensive resource for systematical analysis of immune infiltrates across diverse cancer types. The relationship between the expression of RIPK2 and immune cells infiltration was analyzed through “gene” module across human 33 cancers. Immune score, stromal score and ESTIMATE score were valuable in predicting the prognosis of cancer patients (Becht et al. 2016; Yoshihara et al. 2013). We calculated immune score, stromal score and ESTIMATE score of each sample by the R software package ESTIMATE (https://bioinformatics.mdanderson.org/estimate/rpackage) based on TGGA Pan-Cancer data. The R package psych (version 2.1.6) was used to analyze the correlation between RIPK2 with immune score, stromal score and ESTIMATE score. Pearson correlation analysis was performed to calculate the correlation coefficient between the two factors.

### RIPK2 and immunotherapy response

Tumor immune dysfunction and exclusion (TIDE) database (http://tide.dfci.harvard.edu) is a computational framework for immunotherapy response prediction (Jiang et al. 2018). Liu2019_PD1 SKCM cohort and Braun2020_PD1 KIRC cohort were utilized to explore the role of RIPK2 in tumor immunotherapy response. We used T cell dysfunction score to assess the association between the cytotoxic T lymphocyte (CTL) level and overall patient survival with different RIPK2 levels, and exclusion score to describe the gene expression value in T cell exclusion signatures. RIPK2 was compared with other published biomarkers by biomarker evaluation based on their predictive of response outcome and overall survival. The T cell dysfunction score for each gene is defined as the Wald test Z-score, which is the coefficient divided by its standard error. Z-Score standardization can convert data of different metrics into a uniform metric, improving the comparability between data. Differences in the effects of different biomarkers on immunotherapy efficacy were compared by Z-Score.

### Expression level of RIPK2 at the single-cell level

Tumor immune single-cell hub (TISCH) (http://tisch.comp-genomics.org/) is a comprehensive web resource enabling interactive single-cell transcriptome visualization of tumor microenvironment (Sun et al. 2021). In the “dataset” module, we visualized the expression level of RIPK2 at the single-cell level in the BLCA_GSE145281 dataset, SKCM_GSE72056 dataset, and KIRC_GSE111360 dataset. In addition, the difference of RIPK2 expression in immune cells was investigated between immunotherapy responders and non-responders in the SKCM_GSE120575 dataset.

### Gene set enrichment analysis

The Gene set enrichment analysis (GSEA) method was applied to study the potential mechanisms of RIPK2 in the development of cancer. First, we grouped the SKCM and KIRC samples according to the median expression of RIPK2 in all samples, called the “high” group with the expression greater than the median, and the “low” group with less than the median expression, compared the differences in gene expression between the two groups, and ranked them according to the value of the foldchange. Then we chose the Hallmarker gene sets which were defined based on prior biological knowledge to analyze all samples in GSEA method using the R package clusterProfiler (version 4.0.5). The normalized enrichment score (NES) is the primary statistic for examining gene set enrichment results. By normalizing the enrichment score, GSEA accounts for differences in gene set size and in correlations between gene sets and the expression dataset. The false discovery rate (FDR) is the estimated probability that a gene set with a given NES represents a false positive finding. We chose NES and FDR as the indicators of enrichment, (Gene sets with |NES|>1 and FDR < 0.25 were considered to be enrichment significant) and used the R package ggplot2 (version 3.3.3) to visualize the results.

### RIPK2 and drug response

CellMiner (https://discover.nci.nih.gov/cellminer/) is a database that integrates molecular and pharmacological data for the NCI-60 cancerous cell lines (Reinhold et al. 2012). The correlation between RIPK2 expression and drug response was explored by CellMiner.

### Immunofluorescence staining

Kidney renal clear cell carcinoma tissue array was obtained from Shanghai Zhuoli biotechnology Co.,Ltd (Zhuoli Biotechnology Co, Shanghai, China). The automatic immunohistochemical staining machine (Leica, Bond III, Germany) was used for dewaxing and antigen repair. After five rinses with phosphate-buffered saline (PBS), Tissue array was soaked in hydrogen peroxide solution, incubated at room temperature for 10 min. Then, RIPK2 antibody (Affinity, DF6967, USA, 1:600) was added to the tissue array, incubated at 37 °C for 1 h. After five rinses with PBS, goat anti-rabbit poly-HRP (Leica, DS9800, Germany) was added to the tissue array, incubated at 37 °C for 10 min. The nucleus was stained with DAPI. Finally, 3DHISTECH fluorescence imaging scanner was used for scanning.

### Statistical analysis

All statistical calculations were conducted through R software (version 3.6.3). The comparison of difference between two groups was analyzed using Wilcoxon rank-sum test. The comparison of difference between three groups or more groups was analyzed using the Kruskal–Wallis test.

## Result

### The mRNA expression of RIPK2 in different cancers

**Fig. 1:**
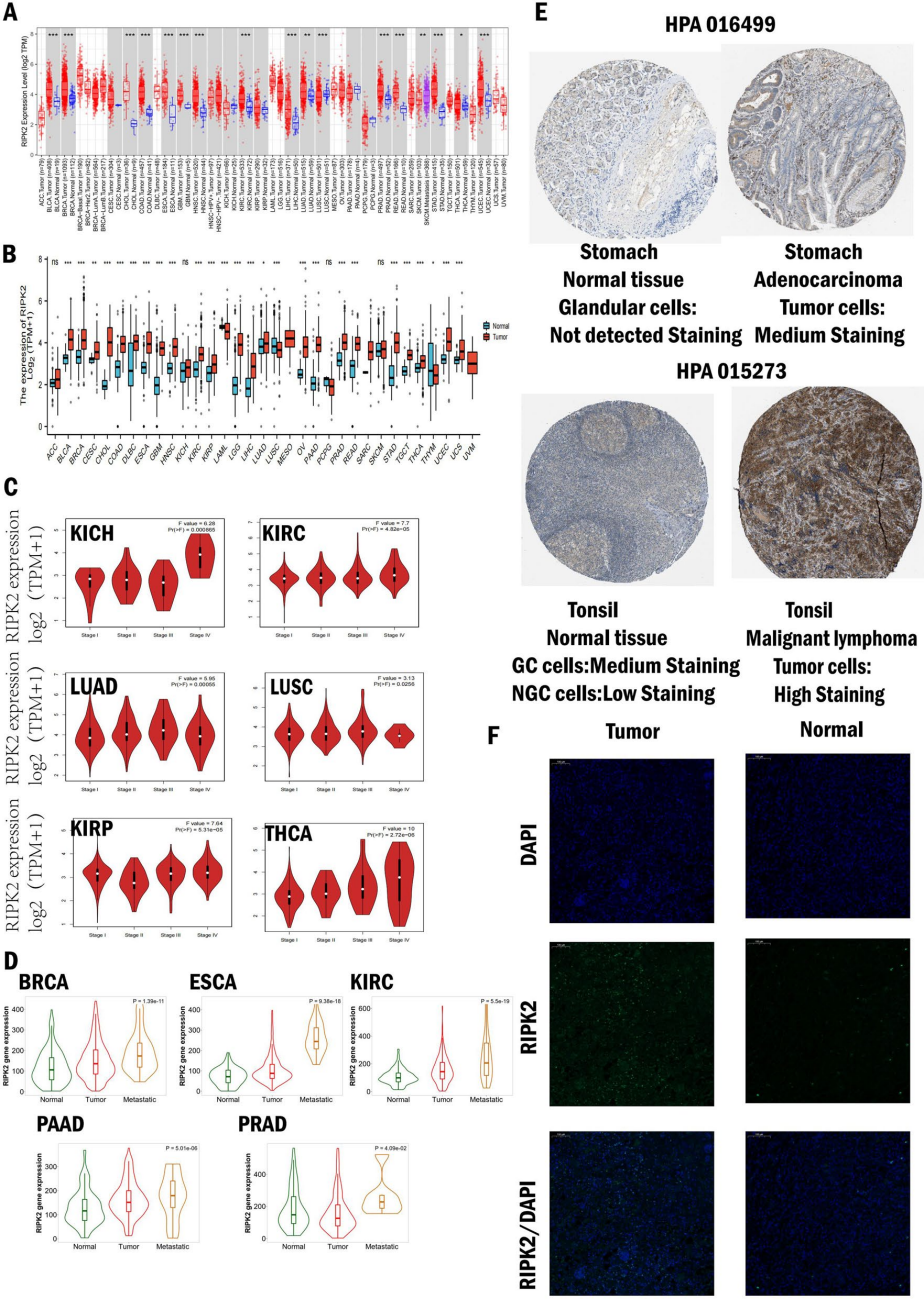
RIPK2 expression in various cancers and different pathological stages. **A** The expression of RIPK2 is compared between tumor tissue and normal tissue using TIMER 2.0 (*p < 0.05, **p < 0.01, and ***p < 0.001). **B** Expression status of RIPK2 in different cancer types from TCGA and GTEx data (*p < 0.05, **p < 0.01, and ***p < 0.001). **C** Based on the TCGA data, RIPK2 expression levels of different types of cancer (KICH, KIRC, KIRP, LUAD, LUSC, THCA) were analyzed by different pathological stages (stage I, stage II, stage III, and stage IV). **D** Different RIPK2 expression levels between normal tissue, tumor tissue, and metastatic tissue in PRAD, BRCA, KIRC, ESCA, and PAAD. **E** Expression of RIPK2 protein in stomach normal tissue, stomach adenocarcinoma, tonsil normal tissue, and tonsil malignant lymphoma from HPA database. **F** Immunofluorescence staining of RIPK2 protein in the normal tissue and tumor tissue in KIRC

We first used the TIMER online network tool, using data from TCGA database, compared the differences in the expression of RIPK2 in tumor tissue and normal tissue in 33 cancer types, and specific information about 33 cancer types were described in Table 1. We found that RIPK2 is widely over-expressed in tumor tissues, such as BLCA, BRCA, CHOL, COAD, ESCA, GBM, HNSC, KIRC, LIHC, LUSV, PRAD, READ, STAD, UCEC (Fig. 1A). Due to the lack of normal control of several tumors in the TCGA database, we analyzed the TCGA and GTEx data. In addition to the above 14 tumors, RIPK2 was significantly higher in DLBC, while lower in LAML (Fig. 1B). Moreover, the relationship between RIPK2 expression and tumor pathological stages was explored, and the results showed that the expression of RIPK2 was significantly correlated with pathological stages (Fig. 1C). Further, the RIPK2 expression of metastatic PRAD, BRCA, KIRC, ESCA, and PAAD tumor was significantly higher than primary tumor **(**Fig. 1D**)**. Immunohistochemistry showed the expression level of RIPK2 protein increased in stomach adenocarcinoma and tonsil malignant lymphoma **(**Fig. 1E**)**. Through the immunofluorescence staining, we found that the expression level of RIPK2 protein in the tumor tissue was higher than the normal tissue in KIRC **(**Fig. 1F**)**. The above results suggested that RIPK2 perhaps played a carcinogenic role in tumor progression, and its clinical value is worth exploring.

**Table 1:** Abbreviations and full names of all TCGA cancers

Abbreviation	Full name
ACC	Adrenocortical carcinoma
BLCA	Bladder urothelial carcinoma
BRCA	Breast invasive carcinoma
CESC	Cervical squamous cell carcinoma and endocervical adenocarcinoma
CHOL	Cholangiocarcinoma
COAD	Colon adenocarcinoma
DLBC	Lymphoid neoplasm diffuse large B-cell lymphoma
ESCA	Esophageal carcinoma
GBM	Glioblastoma multiforme
HNSC	Head and neck squamous cell carcinoma
KICH	Kidney chromophobe
KIRC	Kidney renal clear cell carcinoma
KIRP	Kidney renal papillary cell carcinoma
LAML	Acute myeloid leukemia
LGG	Brain lower grade glioma
LIHC	Liver hepatocellular carcinoma
LUAD	Lung adenocarcinoma
LUSC	Lung squamous cell carcinoma
MESO	Mesothelioma
OV	Ovarian serous cystadenocarcinoma
PAAD	Pancreatic adenocarcinoma
PCPG	Pheochromocytoma and paraganglioma
PRAD	Prostate adenocarcinoma
READ	Rectum adenocarcinoma
SARC	Sarcoma
SKCM	Skin cutaneous melanoma
STAD	Stomach adenocarcinoma
TGCT	Testicular germ cell tumors
THCA	Thyroid carcinoma
THYM	Thymoma
UCEC	Uterine corpus endometrial carcinoma
UCS	Uterine carcinosarcoma
UVM	Uveal melanoma

### The prognostic value of RIPK2 expression

**Fig. 2:**
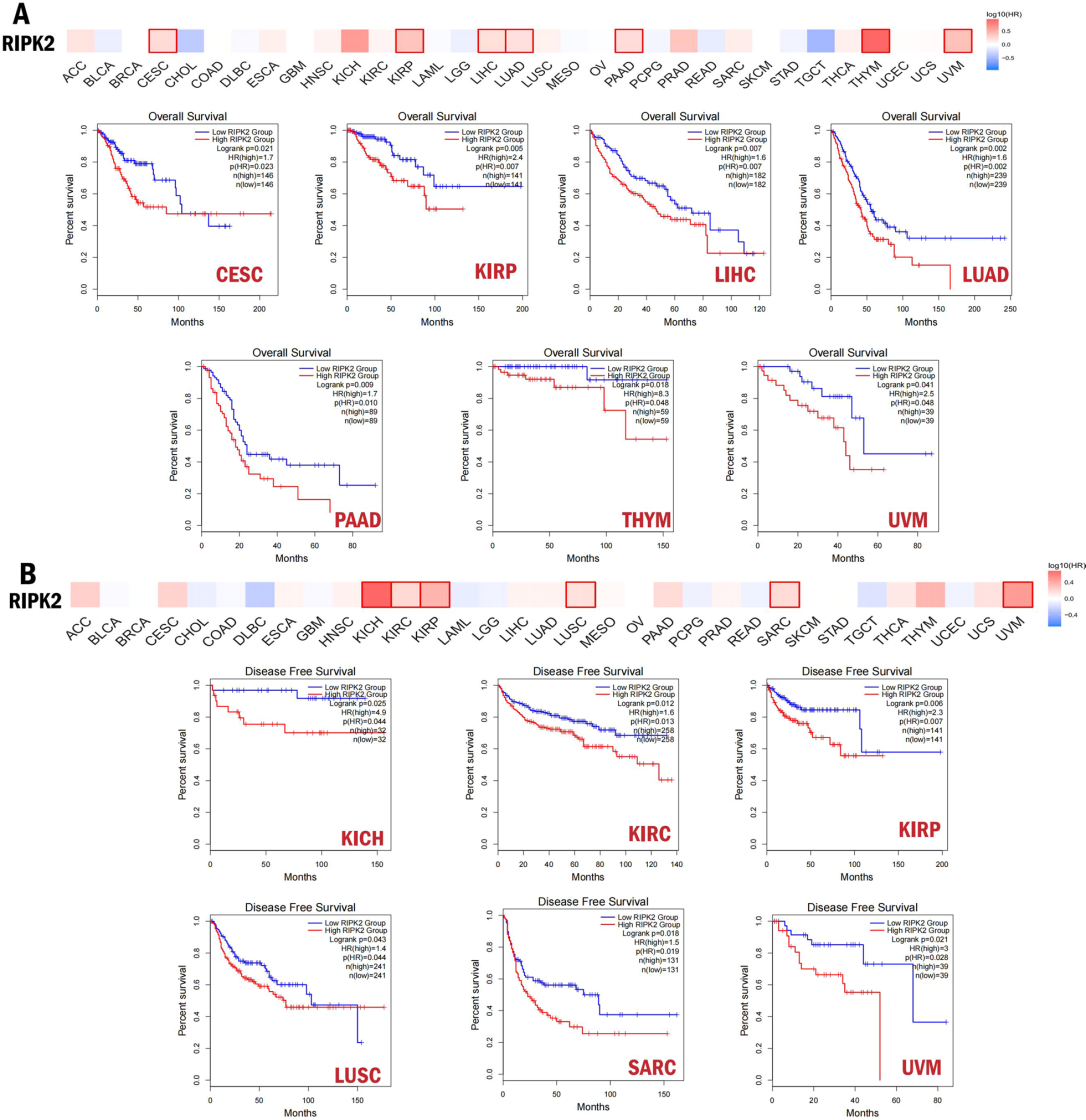
Correlation between RIPK2 expression and overall survival and disease-free survival of patients in different kinds of tumor. **A** Survival map (OS) from the online tool GEPIA2 (upper panel). Kaplan–Meier survival plots (OS) comparing high and low expression of RIPK2 in different kinds of cancer (CESC, KIRP, LIHC, LUAD, PAAD, THYM, UVM) (lower panel). **B** Survival map (DFS) from the online tool GEPIA (upper panel). Kaplan–Meier survival plots (DFS) comparing high and low expression of RIPK2 in different kinds of cancer (KICH, KIRC, KIRP, LUSC, SARC, UVM) (lower panel)

In order to explore the predictive value of RIPK2 in human cancer, GEPIA2 online tool was used to compare the prognostic difference between RIPK2 high expression group and low expression group. We found that the OS of RIPK2 high expression group was shorter than that in the low expression group in CESC (HR = 1.7, p = 0.021), KIRP (HR = 2.4, p = 0.005), LIHC (HR = 1.6, p = 0.007), LUAD (HR = 1.7, p = 0.002), PAAD (HR = 1.7, p = 0.09), THYM (HR = 8.3, p = 0.018), UVM (HR = 2.5, p = 0.041) (Fig. 2A). Meanwhile, higher expression of RIPK2 was associated with shorter DFS in KICH (HR = 4.9, p = 0.025), KIRC (HR = 1.6, p = 0.012), KIRP (HR = 2.3, p = 0.006), LUSC (HR = 1.4, p = 0.043), SARC (HR = 1.5, p = 0.018), UVM (HR = 3, p = 0.021) (Fig. 2B). These results showed that the expression of RIPK2 was often related to the poor prognosis of patients, and further confirmed that RIPK2 played an important role in the occurrence and development of various cancers.

### Genetic alteration analysis of RIPK2 in different cancers

**Fig. 3:**
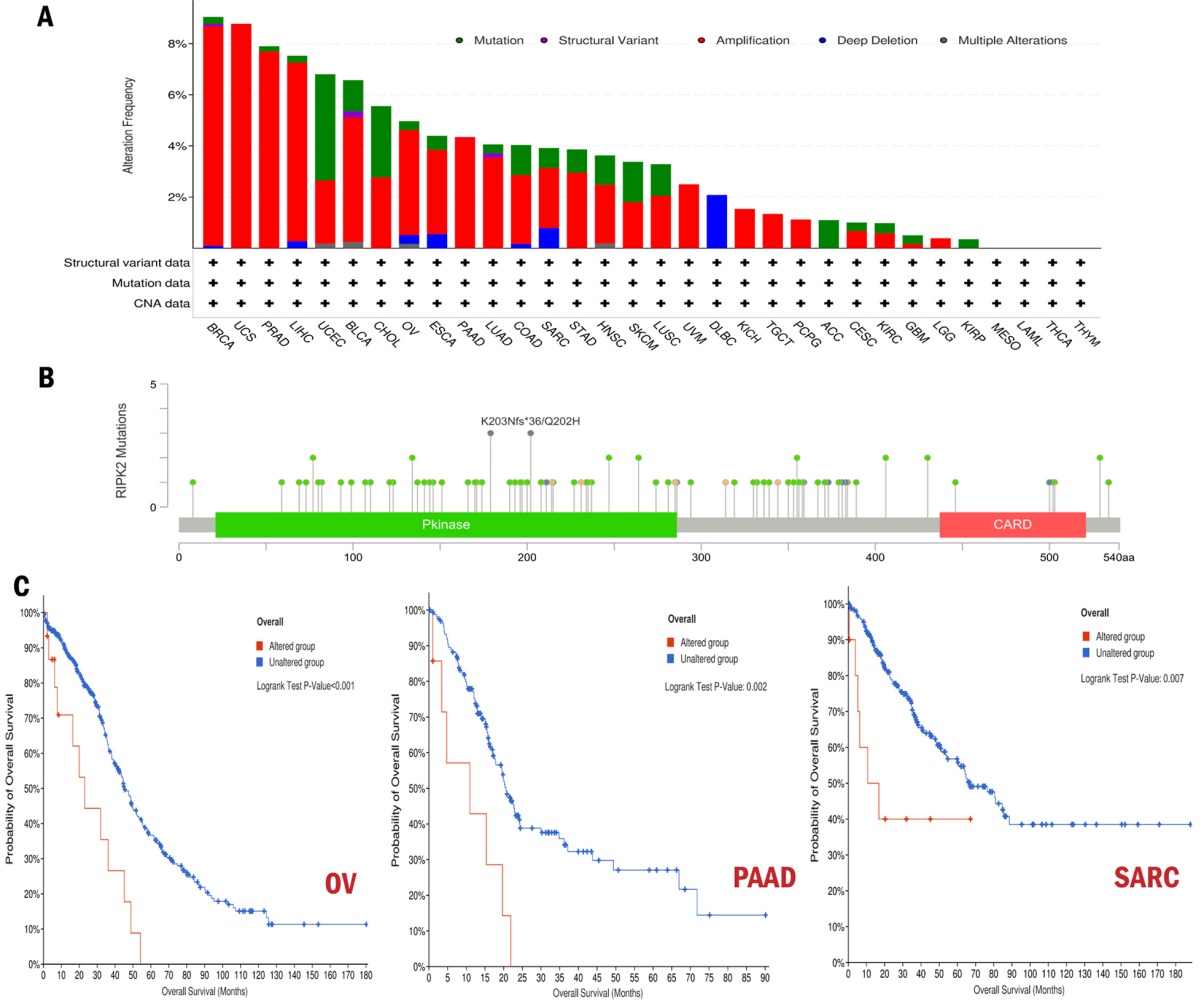
Genetic alteration analysis of RIPK2 in different cancers. **A** Genetic alteration frequency and type of RIPK2. **B** RIPK2 mutation sites in TCGA samples. **C** Kaplan–Meier survival plots (OS) comparing altered and unaltered group in OV, SARC, and PAAD

The genetic alteration of RIPK2 in different cancers was investigated through cBioPortal, and we found that this genetic alteration was most pronounced in BRCA. The type of genetic alteration in most tumors was gene amplification. At the same time, we found that the high alteration rate of RIPK2 emerged in BRCA, UCS, and PRAD, while amplification was the most common type and structural variant was the rarest (Fig. 3A). We investigated mutation sites of RIPK2 in TCGA samples **(**Fig. 3B**)**. In addition, we explored the relationship between RIPK2 alteration and clinical outcome. We found that the OS of altered group was significantly lower than unaltered group in OV (p < 0.001), PAAD (p = 0.002) and SARC (p = 0.007), which suggested that RIPK2 alteration was often associated with poor prognosis (Fig. 3C).

### Correlation between RIPK2 expression and immune infiltrating level

**Fig. 4:**
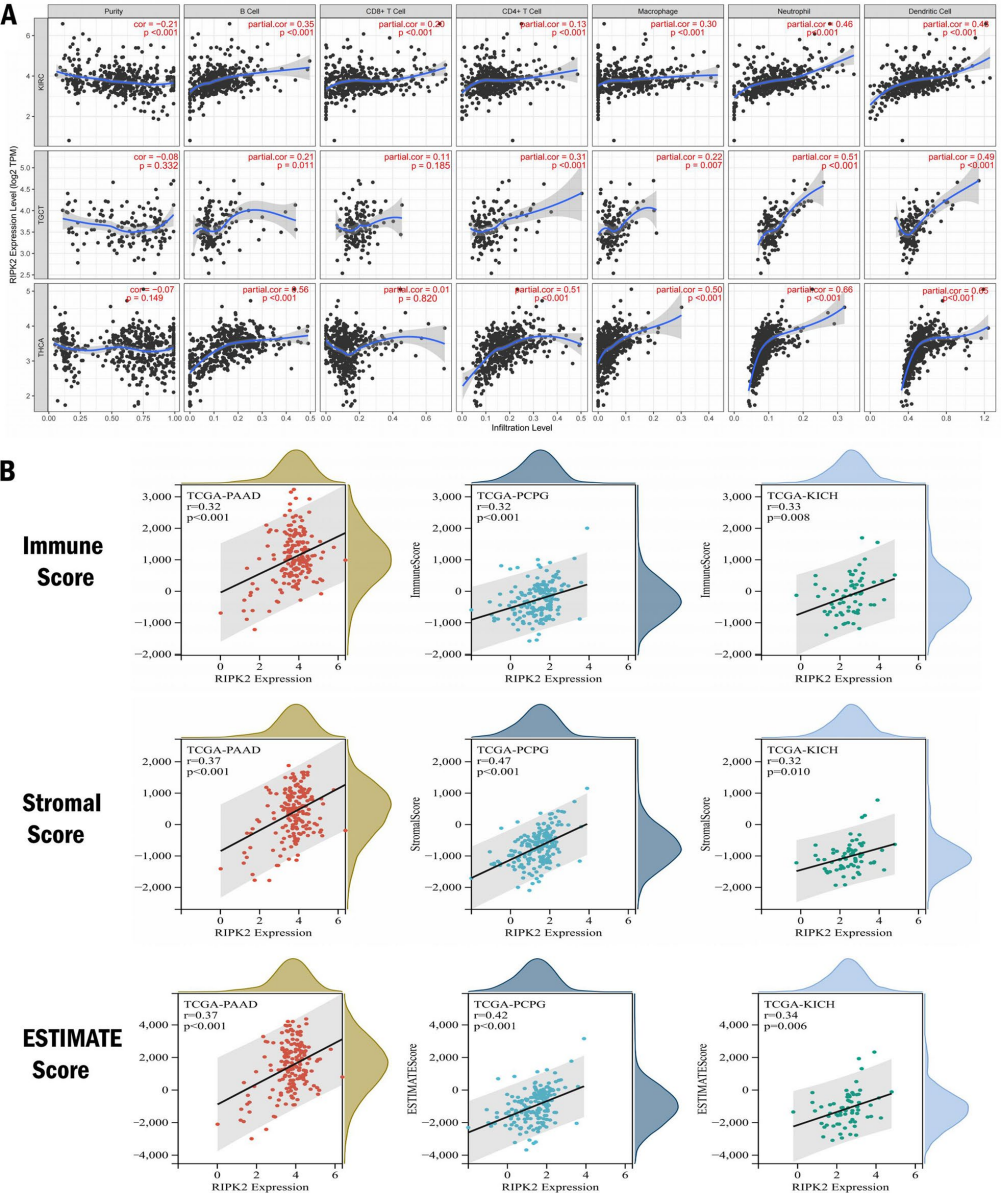
Correlation between the expression of RIPK2 and the level of immune cell infiltration in various types of cancers. **A** Correlation of RIPK2 expression with immune infiltration status in KIRC, TGCT, THCA. **B** Correlation of RIPK2 expression with immune score, stromal score and ESTIMATE score in PAAD, PCPG and KICH

Tumor tissue is composed of various types of cells, including stromal cells, fibroblasts, and immune cells. These cells constitute the tumor’s microenvironment. Therefore, we focused on the correlation between the expression of RIPK2 and the level of immune cell infiltration. RIPK2 was found to promote a series of immune cell infiltration, especially in KIRC, TGCT and THCA. B cells, macrophages, and neutrophils were significantly positively correlated with the expression of RIPK2. In KIRC, the expression of RIPK2 was negatively related to tumor purity (r = − 0.21, p < 0.001), B cell (r = 0.35, p < 0.001), macrophage (r = 0.30, p < 0.001), and neutrophil (r = 0.46, p < 0.001). In TGCT, the expression of RIPK2 was negatively related to B cell (r = 0.21, p = 0.011), macrophage (r = 0.22, p = 0.007), and neutrophil (r = 0.51, p < 0.001). In THCA, the expression of RIPK2 was negatively related to, B cell (r = 0.56, p < 0.001), macrophage (r = 0.50, p < 0.001), and neutrophil (r = 0.66, p < 0.001) (Fig. 4A). In addition, we found that RIPK2 significantly enhanced the immune score, stromal score and ESTIMATE score in PAAD, PCPG and KICH (Fig. 4B). All these data suggested that RIPK2 was closely related to tumor immune infiltration.

### Correlations between RIPK2 expression and immunotherapy response

**Fig. 5:**
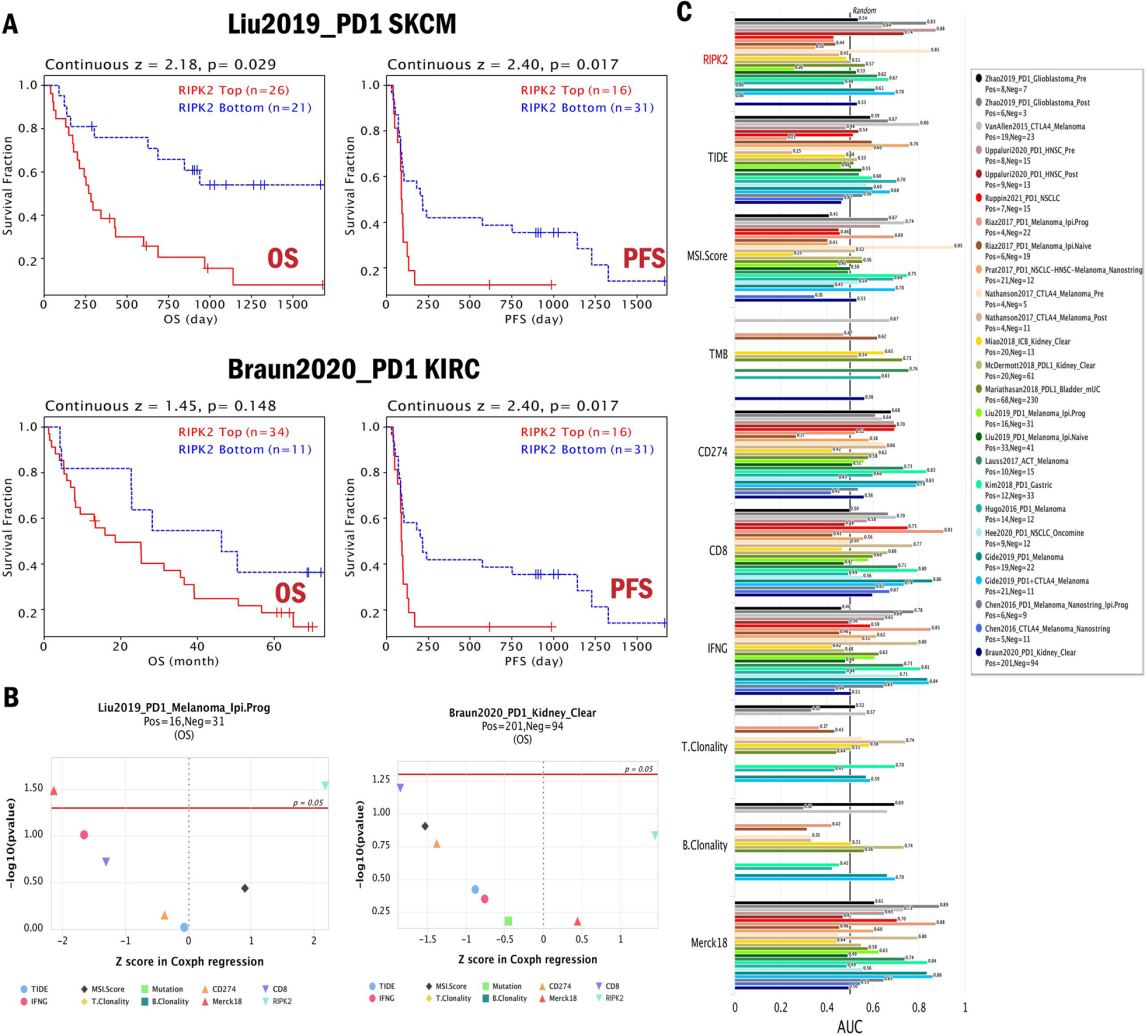
Correlations between RIPK2 expression and immunotherapy response. **A** Kaplan–Meier survival plots (OS) comparing high and low expression of RIPK2 in SKCM and KIRC cohort studies treated with anti-PD1 drug. **B** The plot showed the prognostic value of RIPK2 versus standardized biomarkers in SKCM and KIRC cohort. **C** The bar chart showed the correlation between RIPK2 and standardized biomarkers in the immunotherapy cohorts. The area under the recipient’s working characteristic curve (AUC) was used to evaluate the predictive performance of the test biomarker to the immunotherapy response state

Immunotherapy based on immune checkpoint blocker strengthens the body’s anti-tumor immune response, but only a small percentage of patients respond to treatment. Exploring sensitive and stable immunotherapy response biomarker is beneficial to selecting suitable treatment methods in clinical practice. We explored the relationship between RIPK2 and immunotherapy response through the TIDE database. We found that the high expression of RIPK2 was associated with poor prognosis, and RIPK2 significantly affected the efficacy of immune checkpoint blockade (anti-PD1), reducing OS (z = 2.18, p = 0.029) and progression-free survival (PFS) (z = 2.40, p = 0.017) of patients in Liu2019_PD1 SKCM cohort and PFS (z = 1.98, p = 0.047) of patients in Braun2020_PD1 KIRC cohort **(**Fig. 5A, B**)**. Compared to standardized biomarkers, RIPK2 was more accurate and valuable in predicting immunotherapy outcome for patients with GBM (Zhao2019_PD1_ Glioblastoma, AUC = 0.83) or HNSC (Uppaluri2020_PD1_HNSC, AUC = 0.88) **(**Fig. 5C**)**. These results revealed that RIPK2 was involved in anti-tumor immune response and promoted immunotherapy resistance.

### RIPK2 aggravated CTL dysfunction to promote immunotherapy resistance

**Fig. 6:**
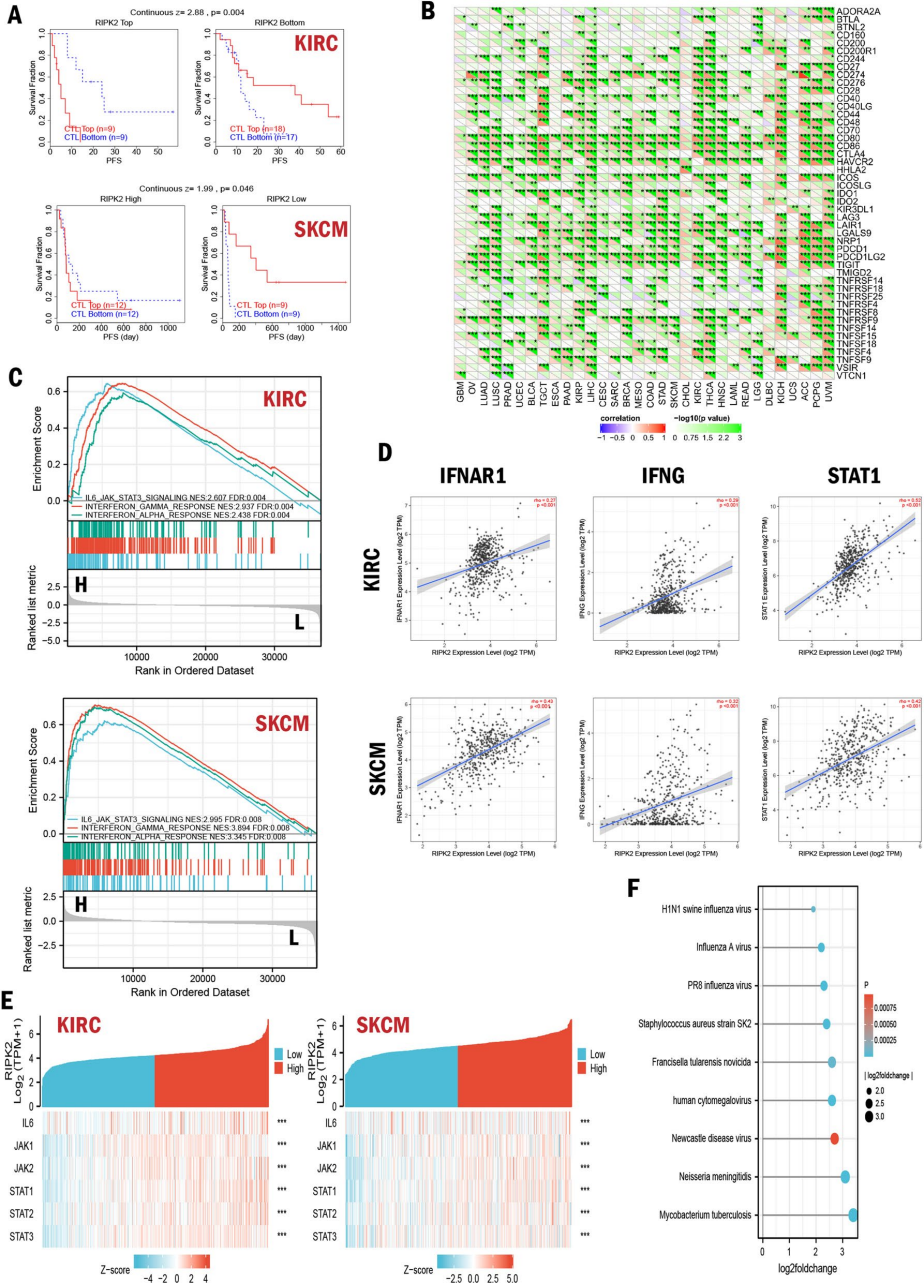
Mechanisms of RIPK2 promoting immunotherapy resistance. **A** The relationship between RIPK2 and CTL dysfunction. **B** The correlation of RIPK2 and known immune checkpoints marker through all TCGA cancers (*p < 0.05, **p < 0.01, and ***p < 0.001). **C** GSEA analysis for SKCM and KIRC samples with high RIPK2 expression and low expression. **D** The correlation of RIPK2 and IFNG, STAT1, IFNAR1 in SKCM and KIRC. **E** The correlation of RIPK2 and the key components of IL-6/JAK/STAT3 signaling. **F** Change of RIPK2 expression after pathogen infection

Cytotoxic T lymphocyte plays a major role in the anti-tumor immune, and CTL dysfunction promotes tumor immune escape and immunotherapy resistance. TIDE database was used to evaluate the relationship between RIPK2 and CTL dysfunction. We found that the expression of RIPK2 was positively correlated with CTL dysfunction level in KIRC (z = 2.88, p = 0.004), SKCM (z = 1.99, p = 0.046). When the expression level of RIPK2 was low, CTL infiltrating was beneficial for the long-term survival of patients with KIRC or SKCM, but the high expression level of RIPK2 weakened or even reversed the beneficial effect **(**Fig. 6A**)**. Gene co-expression analysis indicated that RIPK2 was positively correlated with the expression of immune checkpoint markers, such as PDCD1 (PD-1), CD274 (PD-L1), CTLA4 and TIGIT, which suggested that the expression of RIPK2 was related to the immune escape and immunotherapy responses **(**Fig. 6B**)**. GSEA analysis showed that RIPK2 significantly improved IL-6/JAK/STAT3 signaling (NES = 2.995, FDR = 0.008), interferon-gamma response (NES = 3.894, FDR = 0.008) and interferon-alpha response (NES = 3.345, FDR = 0.008) in SKCM, which was similar to the role in KIRC **(**Fig. 6C**)**. Further, the correlation of RIPK2 to the key components of these pathways that contributed to immunotherapy resistance was observed **(**Fig. 6D, E**)**. Moreover, we found that pathogens infection significantly increased the expression of RIPK2, especially mycobacterium tuberculosis which were prone to immune escape (log FC = 3.4, p < 0.001) **(**Fig. 6F**)**.

### RIPK2 specific expression on monocyte/macrophage

**Fig. 7:**
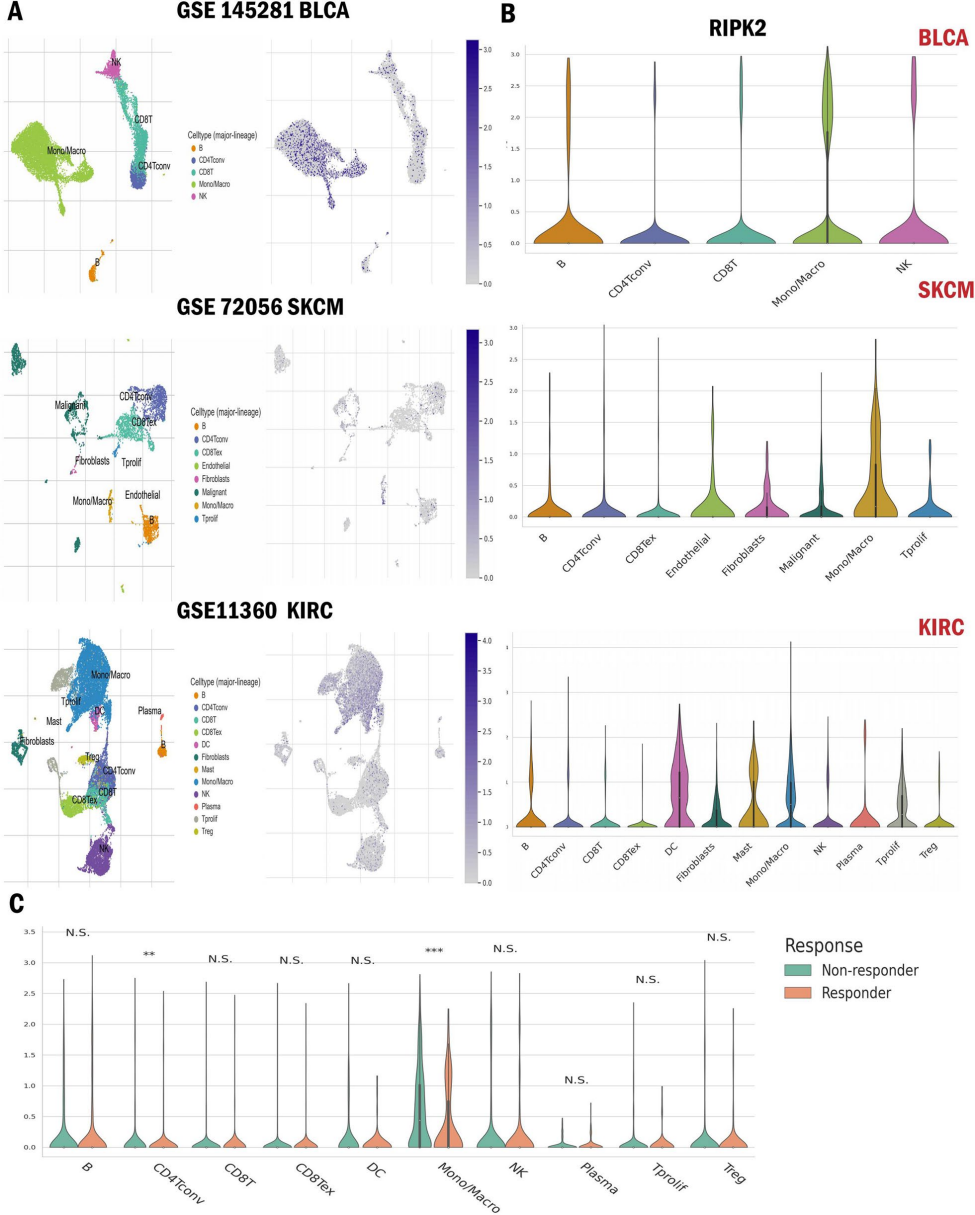
Expression level of RIPK2 at the single-cell stage. **A** UMAP graphs showed cellular clusters and the expression level of RIPK2 in different cellular types of BLCA, SKCM, and KIRC. **B** Single-cell RIPK2 expression profile of different cellular types in BLCA, SKCM, and KIRC. **C** Comparison of single-cell RIPK2 expression profile of immunotherapy non-responder with responder

To explore the potential mechanisms by which RIPK2 affected tumor immune microenvironment, public single-cell RNA-seq (scRNA) datasets were used to analyze the expression level of RIPK2 on different immune cells. We found that RIPK2 were mainly expressed on monocyte/macrophage of (Fig. 7A, B). Interestingly, immunotherapy responder’s monocyte/macrophage had the higher expression of RIPK2 than non-responder, which indicated that RIPK2 was associated with tumor immune suppressive cells and immunotherapy resistance (Fig. 7C).

### Correlation of RIPK2 and drug response

**Fig. 8:**
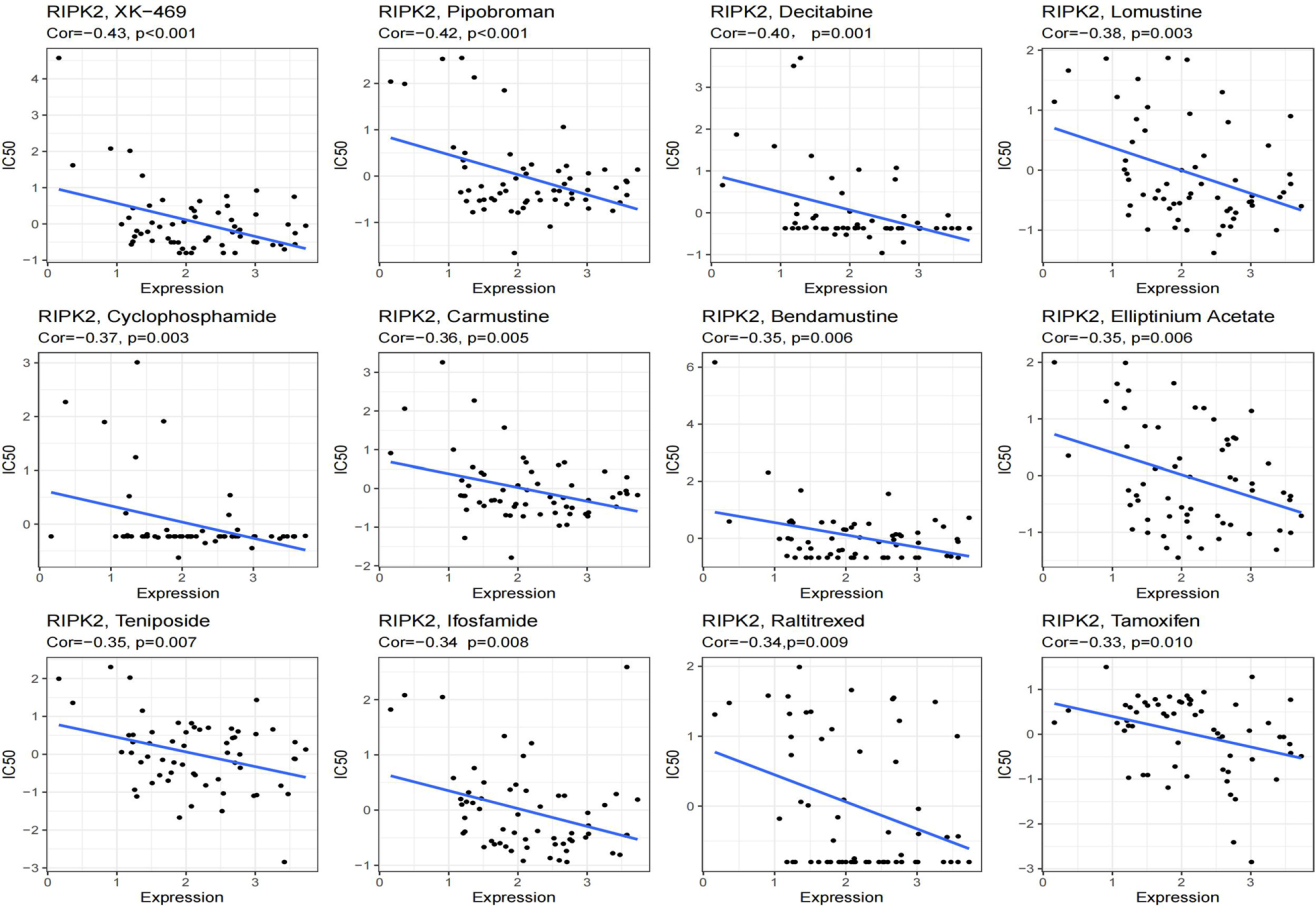
Correlation of RIPK2 and drug response

We analyzed the relationship between the expression of RIPK2 and the drug response in 60 tumor cell lines. The results showed that RIPK2 was significantly negatively correlated with IC50 of 12 drugs (Fig. 8), which provided guidance for the treatment of RIPK2 high expression patients.

## Discussion

RIPK2 with carboxy-terminal caspase activation and recruitment domain (CARD) was indispensable for innate and adaptive immunity (Magalhaes et al. 2011). Innate immune and adaptive immune cells are involved in the development of malignant tumors, and their interaction with cancer cells constitutes an environment that promotes tumor growth and metastasis (Hinshaw and Shevde 2019). In order to comprehensively explore the role of RIPK2 in cancer, we first analyzed the expression of RIPK2 in 33 tumors, and the result showed that RIPK2 was widely overexpressed in tumor tissues compared with normal tissues. Moreover, the expression of RIPK2 was related to tumor classification in several cancers like LUAD, LUSC, THCA and multiple kidney cancers (KICH, KIRC and KIRP). Compared with the low-expression RIPK2 group, the OS and DFS of the high-expression RIPK2 group were found to be shorter through prognosis analysis. Consistent with our result, RIPK2 was reported to affect tumor progression by promoting cellular autophagy and to accurately predict prognosis in PRAD (Li et al. 2020). The association between mutations of genes and the progression and metastasis of human cancers has been investigated by growing researches (Berger and Mardis 2018; Huang et al. 2018). However, just a few alterations significantly impact cancer progression, while the rest of the mutations are trivial (Alexandrov and Stratton 2014). Survival analysis revealed that the alteration of RIPK2 decreased overall time of OV, SARC, and PAAD. Nevertheless, there are no studies about the molecular mechanism by which RIPK2 alteration affect prognosis of human cancers, and our result provided a basis for further in-depth study of the carcinogenic effects of RIPK2.

Systemic reorganization of immune state occurs in the development and progression of diverse malignancies. As a fundamental innate immune response, inflammation affects all stages of the tumor, which provides an effective way to prevent and treat cancer in clinical practice (Elinav et al. 2013). Inhibiting inflammatory responses has the potential to not only prevent or delay the occurrence of cancer, but also to improve the efficacy of conventional therapeutics and immunotherapies (Hou et al. 2021). B cells, macrophages, and neutrophils were significantly positively correlated with the expression of RIPK2. Previous research has shown that B cells or macrophages were associated with poor prognosis in LUAD at early clinical stage (Liu et al. 2017). As the body’s first line of defense against infection, neutrophils respond to various inflammatory signals, including cancer (Giese et al. 2019). Moreover, RIPK2 affected immune score, stromal score and ESTIMATE score for a wide range of cancers. Therefore, the evidence suggested that immune infiltration was a mechanism by which RIPK2 promoted cancer progression.

Immunotherapy that targets the immune system has revolutionized cancer treatment. Regulation of immune system by immune checkpoint blockade (ICB), such as anti-CTLA4, anti-PD1 and anti-PDL1, leads to durable responses in human pan-cancer (Hiam-Galvez et al. 2021). However, most patients do not respond to ICB (primary resistance), and some responders will gradually develop resistant to ICB (acquired resistance) (Sharma et al. 2017). IL-6/JAK/STAT3 signaling strongly inhibits anti-tumor immune response through negative regulatory effects of T cells and natural killer cells, which contributes to the formation of primary immunotherapy resistance (Johnson et al. 2018). Interferon-gamma (IFNG) secreted by cytotoxic T lymphocyte inhibits the function of CTL by increasing the expression of tumor cell PDL1, leading to tumor immune evasion (Spranger et al. 2013). Moreover, IFNG mediates PDL1-independent adaptive resistance through increasing the expression of ligands for multiple T cell inhibitory receptors. STAT1 was the crucial regulatory factor of PDL1-independent adaptive resistance, and knocking out STAT1 led better anti-tumor immune response (Benci et al. 2016). Our research confirmed IL-6/JAK/STAT3 signaling and IFNG response were significantly elevated in the RIPK2 high expression group. Gene co-expression analysis indicated the tight interaction between RIPK2 with PDL1 and STAT1. Based on the above results, we concluded that RIPK2 triggered CTL dysfunction in a variety of ways to promote tumor immune resistance, which was consistent with the result predicted by the TIDE database. Interestingly, pathogen infection also significantly increased the expression of RIPK2, suggesting that RIPK2 may be a universal regulatory factor for immune escape of tumor and pathogen.

Even though we have consolidated and analyzed pan-cancer information from various databases, this study still had several limitations. First, despite bioinformatics analysis provided us a preliminary insight of the crucial role of RIPK2 in cancer progression and immunotherapy resistance, biological experiments in vitro and in vivo are still needed to validate our findings and promote clinical transformation. Further mechanism studies will contribute to determine the precise molecular function of RIPK2 in tumorigenesis. Second, the role of RIPK2 in different cancers is heterogeneous, and the causes of heterogeneity still have to be explored in depth, which can help with accurate and personalized treatment of cancer.

## Conclusions

In conclusion, to our knowledge we conducted the first comprehensive pan-cancer study of RIPK2. The result of pan-cancer analysis showed that RIPK2 was overexpressed in tumor tissues and served as a promising prognostic marker in multiple cancers. Further analysis suggested that RIPK2 promoted immune therapy resistance via triggering cytotoxic T lymphocytes dysfunction. Through our research, we emphasized the promising value of RIPK2 in immunotherapy and provided new idea and therapeutic target for future research.

## Data Availability

All data generated or analyzed during this study are publicly available. The TCGA and GTEx data can be downloaded in UCSC XENA website (https://xenabrowser.net/datapages/). The immunotherapy cohorts can be found in TIDE database (http://tide.dfci.harvard.edu). The pharmacological data for the NCI-60 cancerous cell lines can be downloaded in CellMiner database (https://discover.nci.nih.gov/cellminer/).

## Abbreviations

RIPK2: Receptor-interacting protein kinase 2
SKCM: Skin cutaneous melanoma
KIRC: Kidney renal clear cell carcinoma
TCGA: The Cancer Genome Atlas
GTEx: Genotype-Tissue Expression
HPA: Human Protein Atlas
GEPIA2: Gene Expression Profiling Interactive Analysis 2
TIMER: Tumor immune estimation resource
OS: Overall survival
DFS: Disease-free survival
HR: Hazard ratio
TIDE: Tumor immune dysfunction and exclusion
CTL: Cytotoxic T lymphocyte
TISCH: Tumor Immune Single-cell Hub
GSEA: Gene set enrichment analysis
NES: Normalized enrichment score
FDR: False discovery rate
PFS: Progression-free survival
CARD: Carboxy-terminal caspase activation and recruitment domain
ICB: Immune checkpoint blockade
IFNG: Interferon-gamma
